# Post-Treatment Edema after Meningioma Radiosurgery is a Predictable Complication

**DOI:** 10.7759/cureus.605

**Published:** 2016-05-09

**Authors:** Alfredo Conti, Antonio Pontoriero, Francesca Siddi, Giuseppe Iatì, Salvatore Cardali, Filippo F Angileri, Francesca Granata, Stefano Pergolizzi, Antonino Germanò, Francesco Tomasello

**Affiliations:** 1 Department of Neurological Surgery, University of Messina; 2 Radiation Oncology, University of Messina; 3 Neuroradiology, University of Messina

**Keywords:** brain edema, meningioma, stereotactic radiosurgery, cyberknife, brain tumor interface, vascular-endothelial growth factor

## Abstract

Symptomatic post-treatment edema (PTE) causing seizures, focal deficits, and intracranial hypertension is a rather common complication of meningioma radiosurgery. Factors associated to the occurrence of PTE still needs to be clarified. We retrospectively analyzed our patients’ data to identify factors associated with the development of symptomatic PTE. Supposed risk factors were systematically analyzed.

Between July 2007 and March 2014, 245 meningiomas in 229 patients were treated by a single fraction or multisession radiosurgery (2-5 fractions) or hypofractionated stereotactic radiotherapy (6-15 fractions) using the CyberKnife system (Accuray Inc., Sunnyvale, CA) at the University Hospital of Messina, Italy.

Local tumor control was achieved in 200 of 212 patients with World Health Organization (WHO) Grade I meningiomas (94%) at a mean follow-up of 62 months. Symptomatic PTE on MRI was diagnosed in 19 patients (8.3%) causing seizure (n=17, 89%), aggravating headache (n=12, 63%), or focal deficits (n=13, 68%). Four variables were found to be associated with the likelihood of edema development, including tumor volume > 4.5 mL, non-basal tumor location, tight brain/tumor interface, and atypical histology. Nonetheless, when multivariate logistic regression analysis was performed, only tumor volume and brain-tumor interface turned out to be independent predictors of PTE development.

Our results suggest that the factor associated with the risk of developing PTE is associated to characteristics of meningioma rather than to the treatment modality used. Accordingly, an appropriate patient selection is the way to achieve safe treatment and long-term disease control.

## Introduction

Stereotactic radiosurgery (SRS) has progressively emerged as both an adjuvant treatment modality for residual tumors and an effective primary treatment of properly selected meningiomas. Radiosurgery is virtually noninvasive, but it does carry a risk of radiation-induced complications. For meningiomas, this risk ranges between 3% and 40% [1-2].

Symptomatic post-treatment edema (PTE) causing seizures, focal deficits, and even intracranial hypertension is probably the most common complication in intracranial meningioma radiosurgery, as it occurs in 6-35% of cases [3-8]. Factors associated with the occurrence of this complication have been analyzed in a few studies, but definitive conclusions on the pathophysiology of such complication are still anticipated. Parameters such as tumor volume, location, and radiation dose seem to play a role [4-5, 8-18], but other issues, including the staging of the dose or volume and the role of peritumoral veins, have not been analyzed to date.

Starting from the empirical assumption that there are specific circumstances in which the occurrence of PTE seems to be highly probable, we retrospectively analyzed our patients’ data to identify factors associated with the development of symptomatic PTE. Different supposed risk factors were systematically evaluated in order to identify specific predictors of symptomatic PTE development.

## Materials and methods

### Patients

Between July 2007 and March 2014, 245 meningiomas in 229 patients were treated by single fraction or multisession radiosurgery (2-5 fractions) or hypofractionated stereotactic radiotherapy (6-15 fractions) using the CyberKnife system (Accuray Inc., Sunnyvale, CA) at the University Hospital of Messina, Italy. Clinical, main demographic characteristics of patients and treatment parameters are summarized in Table 1. The study was approved by the local Institutional Review Board: Comitato Etico Interaziendale della Provincia di Messina.

**Table 1:** Demographic and dosimetric characteristics of patients. Values are expressed as median (mean) ± standard deviation. BED: biologically-effective dose.

Demographic and Dosimetric Characteristics of Patients	
Age	58.5 years (range 21-84)
Sex	145 f/ 84 m
Tumor volume	6.29 (9.4) ± 10.4 mL (range )
Prescribed dose	20 (20) ± 6.6 Gy (range 12-45 Gy)
Isodose	78 (76.2) ± 3% (range 62-86)
No. of fractions	3 (3.5) ± 2.3 (range 1-15)
BED	87.5 (91.7) ± 12.5 Gy (range 72-118 Gy)
Mean dose	27 (24.9) ± 8.5 Gy (range 14.2-48 Gy)
Maximal dose	27.8 (27.2) ± 10.2 Gy (range 15-64 Gy)
Conformality index	1.33 (1.4) ± 0.6 (range 1.1-4.3)

### Imaging and treatment

Treatments were delivered using the CyberKnife, an image-guided, frameless, LINAC-based, 6 MV radiosurgery system. The patient’s head was immobilized with a thermoplastic mask for imaging acquisition and treatment. The neuroimaging technique consisted of a thin-section, contrast-enhanced, multiplanar reconstruction-gradient echo volumetric study conducted on a Siemens Magnetom 1.5-T MR imaging system (Siemens, Erlangen, Germany), performed at the following parameters: repetition time (TR) 9.7 ms, echo time (TE) 4 ms, matrix 200 × 256, flip angle 1, orientation sagittal. A multislice, contrast-enhanced, head computerized tomography (CT) was also performed using a multislice scanner, Siemens Sensation 16 (Siemens, Erlangen, Germany). Contouring of the tumor and the critical volumes was performed on the co-registered MR and CT dataset. Manual contouring was done in the axial plane with simultaneous display of contours on reconstructed orthogonal images.

### Selection of doses and fractionations

The selection of the marginal and maximal doses and the number of sessions were influenced by multiple factors including tumor volume, the volume of the irradiated brainstem, optic nerve, or chiasm, and visual function, as well as history of previous radiation therapy. Doses in single fractions ranged from 12.5 - 16 Grays (Gy). For multisession radiosurgery and hypofractionated stereotactic radiotherapy (hSRT), we tried to achieve doses of radiation to the planned target volume (PTV) equivalent to 13.0 Gy in a single fraction (as in single fraction SRS) or 54.0 Gy in 30 fractions (as with conventionally fractionated radiotherapy). The equivalence was obtained through the linear-quadratic model. According to this model, the biologically effective dose (BED) is an approximate quantity by which different radiotherapy fractionation regimens may be compared. For a regimen employing n equal fractions, the BED will be:

BED = E/α = nD (1 + D /α/β)

where n = number of fractions, D = dose/fraction, and nD = total dose.

According to previous personal evaluation and radiobiological modeling, for meningiomas, we calculated an α/β equal to 2 Gy [19].

An inverse planning algorithm using a nonisocentric technique determined the optimal treatment planning program. The ray-tracing algorithm was routinely used for this purpose. Some of the methods utilized included: 1) selection of the size and number of collimators, balancing the necessity of coverage, reduction of the number of radiation beams, and monitor units with the necessity of steep dose gradients in specific areas; 2) the addition of tuning structures to reduce uncontrolled dose diffusion; 3) definition of dose constraints and their weight to the target volume and critical structures; and 4) maximization of resolution of dose calculation using the smallest calculation grid and calculation grid expansion to evaluate distant isodose distribution.

### Patient assessment

All patients underwent serial neurological examinations. The radiological assessment consisted of a contrast-enhanced MRI scan obtained at three months and then every six months for two years followed by yearly scans thereafter. Follow-up MRI studies consisted of contrast-enhanced T1 and T2-weighted, proton density and fluid-attenuated inversion recovery (FLAIR) sequences in all cases. For parasellar meningiomas, endocrinological and ophthalmological examinations were also obtained. Ophthalmological follow-up consisted of visual acuity studies and computerized visual field perimetry testing. Endocrinological follow-up entailed the assessment of thyroid hormones, prolactin, cortisol, and dehydroepiandrosterone (DHEA); IGF-1 serum levels were obtained as well.

### Statistical data analysis and parameters

Descriptive statistics were used to analyze demographic and radiology findings. For the purpose of statistical analysis, variables were categorized as specified below. Contingency tables (Fisher’s exact test) were used to compare categorical variables in the univariate analysis. To perform univariate analysis the INSTAT 3.0 software (GRAPHPAD, San Diego, CA) application was used.

Multivariate analysis was performed using the multiple logistic regression method. Variables which were statistically significant in the univariate analysis were transformed into binary variables to be used in the logistic regression model. For non-dichotomic variables, cut-off values were chosen on the base of clinical criteria and literature data and are detailed in Table 2. For instance, we choose BED values above 94 Gy as the threshold as this was considered clinically relevant, independently of the median or mean BED values of the whole series. Similarly, we chose clinically relevant cut-off values for all radiobiological factors (prescribed dose, isodose line, mean and maximal dose, and conformality index). The cut-off value for tumor volume was chosen on the basis of the literature data. The brain/tumor interface was dichotomized into a “smooth” type, in which the tumor was well demarcated from the brain by a small, preserved subarachnoid space that was visible on MRI, and a “tight” type if the MRI suggested a direct contact between the tumor surface and the cortical or subcortical white matter. This tight type of interface was also suggested by the presence of perilesional edema. To perform multivariate analysis, the STATCALC 7.1.1 software (AcaStat, Poinciana, Fl) was used. P < 0.05 was considered statistically significant.

## Results

Two hundred and forty-five intracranial meningiomas in 229 patients, with at least a two-year follow-up, were included in this analysis. The mean age of patients at the time of radiosurgery was 58.5 years (range: 24-84 yrs). One hundred and forty-five (63%) of the 229 patients were women. Thirty-four (15%) patients had a preexisting stable focal deficit.

**Figure 1:**
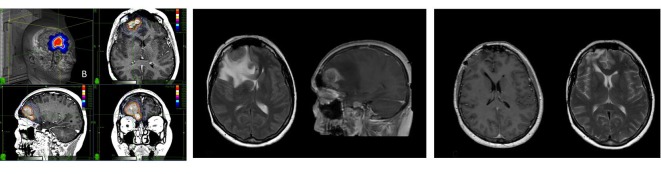
Single fraction CyberKnife radiosurgery treatment of a right frontal meningioma causing symptomatic post-treatment edema (PTE) with resolution after surgical resection of the tumor. Left: Right frontal meningioma radiosurgery treatment plan (single fraction; prescribed dose 13 Gy) in a patient affected by meningiomatosis. The patient had undergone 5-session (25 Gy) radiosurgery for a petroclival meningioma one year before. Middle: Seven months after the treatment, the patient presented with confusion and seizures progressing to status epilepticus. The MRI showed perilesional edema causing frontal lobe compression with midline shift. Right: The patient underwent resection of the frontal meningioma with quick resolution of edema and symptoms. Of note, the treatment of the skull base tumor did not cause any complication.

A previous surgical resection had been performed in 168 of 245 tumors (68.6%). Twenty tumors in this series were confirmed to be atypical (WHO Grade II) on histological examination. Moreover, 21 (9%) tumors had been treated previously with conventional fractionated radiotherapy or single fraction radiosurgery. Symptomatic PTE on MRI was diagnosed in 19 patients (8%) causing seizure (n=17, 89%), headache (n=12, 63%), or focal deficit (n=13, 68%). The median time to onset of PTE was seven months (range: 4-11 mo). No patient developed symptomatic edema later than 12 months after SRS. All patients with PTE were typically started on oral dexamethasone (8 mg every 12 hours); subsequent dosing was titrated to symptoms and neurological deficits. Anticonvulsants included levetiracetam up to 2500 mg, phenobarbital up to 150 mg, and carbamazepine up to 800 mg alone by itself, or variably combined. Medical treatment achieved symptoms control within 30 days in eight (42%) of 19 patients. Patients who did not satisfactorily respond to drugs underwent surgical resection of the tumor (Figure 1). Eleven patients underwent a craniotomy and tumor resection with immediate resolution of edema and symptoms. No patient with WHO Grade I meningiomas reported a permanent deficit attributable to prolonged PTE. Local tumor control was achieved in 200 of 212 patients with WHO Grade I meningiomas (94%) at a mean follow-up of 60 months. No patient died because of tumor progression. Among 20 patients with WHO Grade II meningioma, all had local or distant tumor progression at a median follow-up of 58 months. The two-year overall survival was 80%, the four-year overall survival was 14%.

Table 1 depicts patient, tumor, and radiosurgical characteristics of patients. Table 2 shows the association of these and other factors, potentially affecting outcome, with the development of symptomatic PTE. Table 2 also summarizes variables included in the statistical analysis. To perform the statistical analysis, each variable was dichotomized. For non-dichotomic variables, cut-off values were chosen on the basis of clinical criteria and literature data and are detailed in Table 2. 

**Table 2:** Predictors of development of post-treatment edema. Abbreviations: N.S.: non-significant; y/n: yes/not; BED: biological effective dose; WHO: World Health Organization. *Brain/tumor interface type: in the smooth type, the tumor was well demarcated from the brain by preserved subarachnoid space. In the tight type, the MRI suggested a direct contact between the tumor surface and the cortical or subcortical white matter.

Variable	Univariate	Multivariate (Odds Ratio)
Age	N.S.	
Sex	N.S.	
Tumor volume (≤/> 4.5mL)	0.005	0.04 (0.3)
Prescribed dose (≤/> 27.5 Gy)	N.S.	
Prescription isodose (≤/> 75%)	N.S.	
Fractions (single/multiple)	N.S.	
BED (≤/> 94 Gy)	N.S.	
Mean dose (≤/> 27.5 Gy)	N.S.	
Maximal dose (≤/> 30 Gy)	N.S.	
Conformality index (≤/> 1.2)	N.S.	
Histology (WHO I/II)	<0.001	
Tumor location (basal/non-basal)	<0.001	
Previous surgery (y/n)	N.S.	
Pre-existing edema (y/n)	N.S.	
Brain/tumor interface (smooth/tight*)	<0.001	<0.001 (338)
Previous radiation therapy (y/n)	N.S.	

Per the univariate analysis, four variables were found to be associated with the likelihood of edema development, including tumor volume > 4.5 mL, non-basal tumor location, tight brain/tumor interface, and atypical histology (Table 1). No patient with a skull base meningioma developed symptomatic PTE. Only two patients with posterior fossa tumors, who were also affected by multiple sclerosis, presented with parenchymal lesions with hyperintense signal on FLAIR and T2-weighted MR imaging resembling PTE. Nevertheless, none of the patients had symptoms, and hyperintense signals disappeared within six months and were associated with a remarkable shrinkage of the meningioma.

According to our results, a parasagittal/convexity location was associated with an increased risk of PTE (Figure 1). Nonetheless, this association was not present when we considered only previously operated tumors. Operated meningiomas, indeed, turned out to be associated with a lesser risk of PTE independently on the location.

When multivariate logistic regression analysis was performed, only tumor volume and brain/tumor interface turned out to be independent predictors of PTE development (Table 2). We also found that all patients with meningiomas with a parasagittal location, no previous surgery, and an atypical histology (WHO Grade II) developed symptomatic PTE. Also, meningiomas with larger convexity, which were not previously operated upon and with adherent brain/tumor interface, were also associated with a > 90% risk of developing symptomatic PTE in our series.

## Discussion

**Figure 2:**
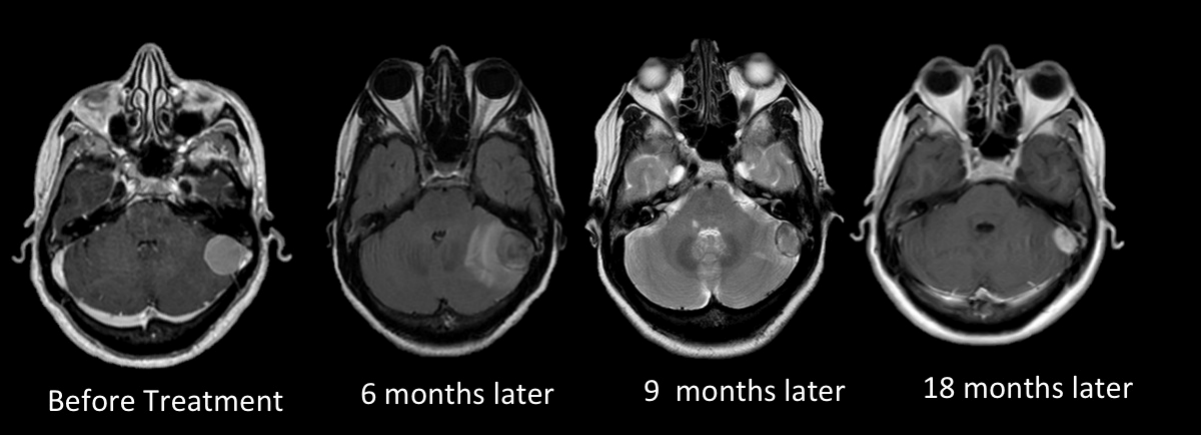
Multisession CyberKnife radiosurgery for a petrosal meningioma in a patient affected by multiple sclerosis. A posterior petrosal meningioma (tumor volume 7.8 mL) in a female patient affected also by multiple sclerosis was treated by multisession radiosurgery (prescribed dose 20 Gy in 3 fractions). Six months after treatment, peritumoral cerebellar hyperintense signal changes appeared on T2 weighed and FLAIR MRI imaging. Symptoms were, however, limited to slight cephalalgia. Low dose corticosteroids were administered to the patient for 3 months. Nine months after treatment, the perilesional signal change disappeared and, at the later imaging confirming control, the tumor showed a remarkable shrinkage.

Our results suggest that larger tumors, an atypical histology, a convexity/parasagittal location, and tight brain/tumor interface are factors associated with the risk of developing symptomatic PTE. Among these factors, larger tumor volume and a tight brain/tumor interface turned out to be independent predictors of symptomatic PTE. Furthermore, patients with atypical parasagittal meningiomas without previous treatment had a 100% risk of developing symptomatic PTE. Also, larger convexity meningiomas, not previously operated and with an adherent brain/tumor interface, were also associated, in our series, with a risk of > 90% of developing severe PTE. Noteworthily, no patient with a skull base meningioma developed symptomatic PTE, including those with large and very large lesions. The only cases in which irradiation of skull base meningiomas caused the development of brain edema were two unusual cases of meningiomas of the posterior fossa in patients with multiple sclerosis (Figure 2). In both cases, patients had PTE after treatment that was easily managed with steroids and resolved within six months. A remarkable shrinkage of the tumor was observed in both cases at 12 months.

Our results suggest also that dose staging, or hypofractionation, does not provide sufficient reassurance of the prevention of PTE. In fact, most patients with parasagittal meningiomas that developed PTE had received multisession radiosurgery. The development of PTE was independent of the invasion and consequential irradiation of peritumoral veins, including major sinuses. Indeed, parasagittal tumors were associated with high risk of PTE, whereas none of the patients with tumors involving the transverse sinuses developed PTE.

All patients with symptomatic PTE underwent high-dose steroid administration, but 11 out of 19 patients needed surgical resection with an almost immediate resolution of the PTE and associated symptoms, including drug-resistant seizures. Our observation that the PTE almost immediately declines after meningioma resection unquestionably demonstrates that the factors responsible for the development of edema reside in the irradiated meningioma, therefore, excluding a direct effect of radiotherapy on the peritumoral brain and vasculature, such as that responsible for PTE in arteriovenous malformations. This interpretation also justifies the lack of protective effect of hypofractionation that is, on the other hand, fundamental in preventing radiosurgery-induced complications when dealing with large skull base meningiomas. Indeed, in these latter cases, hypofractionation may prevent direct effects of radiation on critically radiation-sensitive structures, including the brainstem, optic nerves and chiasm, and cranial nerves.

Peritumoral edema in meningiomas is vasogenic, not cytotoxic, and it is asso­ciated with increased intratumoral vascular permeability [12, 20-22]. Vasogenic edema is caused by an increased capillary permeability with extravasation of serum proteins and fluid into the extracellular spaces. It has been shown that irradiated meningiomas present high expression levels of markers of angiogenesis and hypoxia, (vascular endothelial growth factor (VEGF), and hypoxia-inducible fac­tor-1, respectively) that could be associated with the increased vascular permeability of the tumors [5, 23]. In fact, the VEGF pathway may participate in the formation of brain edema in meningiomas by inducing the formation of "leaky" capillaries, resulting in secretion of VEGF-A and plasma to the peritumoral brain tissue.

Nonetheless, if the causes of PTE were all intrinsic to the meningioma and to its response to radiation, i.e. through an increased secretion of VEGF-A, there would be no reason to explain the prevalence of specific locations for the development of edema. Indeed, parasagittal and convexity meningiomas are associated with a significantly higher probability of edema after radiosurgery [11, 22, 24]. There is sufficient data to quantify this risk. The overall complication rate in the series reported by Kondziolka, et al. of 972 patients who underwent Gamma Knife surgery (GKS) was 7.7%, but the morbidity rate for meningiomas with a parasagittal location was 9.7% [1]. The somewhat surprising fact that non-basal meningiomas have a higher rate of complications than basal meningiomas is also confirmed by many other studies. Patil, et al. [8] reported on 102 supratentorial meningiomas treated with CyberKnife SRS and fractionated radiosurgery. In this study, nine (29%) of 31 patients with parasagittal meningiomas developed symptomatic edema. Hoe, et al. [12] investigated the risks and patterns of evolution of PTE for asymptomatic intracranial meningiomas and found that the non-basal location was an independent risk factor for the development of PTE.

Therefore, PTE seems to be induced by the production of chemicals by the irradiated meningioma, but this occurs only in specific circumstances, such as a parasagittal or hemispheric location and, according to our observations, the absence of a previous surgical manipulation. This suggests that a role may be played by the interface between the tumor and the brain.

Cai, et al. [9] suggested that the tumor-brain interface area is a strong predictor for the development of PTE and proposed a mecha­nistic relationship of the tumor-brain interface disruption by virtue of tumor growth (exerted by large tumors). Our results support the role of this factor showing that volume and tumor-brain interface are independent determinants of symptomatic PTE. Actually, skull base meningiomas are typically and almost invariably extra-arachnoidal tumors. Convexity and, in particular, parasagittal meningiomas are often intra-arachnoidal. In the early nineties, our group published the seminal paper on the brain/tumor interface in meningiomas describing three types of interface: 1) smooth type, in which the tumor was well demarcated from the brain by a small preserved subarachnoid space; no peritumoral edema was usually present preoperatively in such cases; 2) transitional type, in which vessels were often entrapped between brain and tumor, and the arachnoid membrane was very thin and extremely adherent to the tumor. The transitional type is associated with various degrees of halo-like peritumoral edema; 3) invasive type is characterized by vessels crossing the brain-tumor interface [25]. The pial membrane is still present and extremely adherent to the tumor in some areas; however, a disruption of the cortical layer is systematically present in other areas in which the white matter is directly in contact with the tumor. This type of interface is associated with the presence of finger-like edema involving the white matter of the affected hemisphere.

Therefore, it appears that the arachnoid membrane can function as a mechanical and biochemical buffer against mediators released from a tumor. Tumor location where tu­mors are more likely to grow below the arachnoidal layer or to directly penetrate this layer (mostly non-basal vs. skull base) are associated with a significantly increased risk of PTE. Another observation that we consider a proof of the role of brain/tumor interface as the major determinant of PTE development is the fact that parasagittal meningiomas are associated with a high risk of PTE only when SRS was the primary treatment. Remnants and small to medium-sized recurrences were not associated to PTE in our series as a result of a looser interface in these tumors.

Peritumoral edema developed within months and reached its greatest extent at 11 months and decreased thereafter over two years after SRS. Symptom onset and its dura­tion approximated to this timeline. Permanent deficits from PTE after SRS treatment of meningiomas have been reported in less than 3% and were rarely disabling [24, 26-27], although exceptional fatal cases have been also reported [28]. In the present study, no patient har­bored sustained neurological symptoms or reported permanent deficit attributable to PTE because patients with severe or worsening symptoms underwent surgical resection with a surprisingly quick recovery from symptoms, including drug-resistant epilepsy.

## Conclusions

This study represents one of the largest clinical series concerning a relevant but still unsolved issue. Radiosurgery treatment will be more and more frequently considered the first line treatment for meningioma henceforth. Our results suggest that patient selection is fundamental to minimize complications. Actually, we found that the risk of PTE was associated with specific characteristics of the tumor rather than to specific treatment parameters. Such biological features influencing the development of PTE included volume, grading, brain/tumor interface, and location. We also suppose that there are other biological characteristics that might be associated with an increased sensitivity to radiation, i.e. in patients with meningiomatosis. Further studies on the pathophysiology of PTE and its treatment are therefore warranted.
